# An education with audit and targeted feedback intervention to de-implement preoperative surgical urine cultures: a multi-center quasi-experimental study

**DOI:** 10.1017/ice.2026.10470

**Published:** 2026-07

**Authors:** Vishala Parmasad, Kalpana Gupta, William J. O’Brien, Dimitri Drekonja, Gio J. Baracco Lira, Sean O’Neil, Carla Amundson, Sehrish Sikandar, Alejandra Morgan, Judith Strymish, Jeffrey Chan, Madisen E. Brown, Marin L. Schweizer

**Affiliations:** 1 University of Wisconsin-Madison, USA; 2 VA Boston Healthcare System, USA; 3 VA Boston Healthcare System Center for Healthcare Organization and Implementation, USA; 4 Minneapolis VAHCS: Minneapolis VA Medical Center, USA; 5 Miami VA Healthcare System, USA; 6 Audie L Murphy Memorial VA Hospital: Audie L Murphy Memorial Veterans’ Hospital, USA; 7 VA Boston Healthcare System: Veterans Affairs Boston Healthcare System. USA; 8 Boston VA Medical Center: VA Boston Healthcare System Jamaica Plain Campus, USA

## Abstract

Our quasi-experimental study at four Veterans Affairs Medical Centers evaluates whether clinician-centered education, audit, and feedback could reduce unnecessary preoperative urine cultures in asymptomatic neurosurgical, orthopedic, and vascular surgery patients. Urine culture rates decreased from 20.7% to 12.9% (*P* < .01), with orthopedic surgery showing the largest improvements (18.7% to 7.4%; *P* < .01).

## Introduction

Preoperative urine culturing in asymptomatic patients undergoing non-urologic surgery is a low-value intervention that increases costs, antimicrobial exposure, and healthcare utilization without improving patient outcomes.^1–3^ This holds true across neurological, orthopedic, and vascular surgeries.^1,2^ Multiple professional organizations recommend against routine preoperative urine cultures. The Infectious Diseases Society of America (IDSA) and Society for Healthcare Epidemiology of America (SHEA) advise against testing for and treating asymptomatic bacteriuria (ASB) in non-urological surgery patients. ^4^ The American College of Surgeons’ (ACS) best practice statement for preoperative assessment of geriatric patients says routine urinalysis is not indicated,^5^ and the ACS National Surgical Quality Improvement Program guideline for catheter-associated urinary tract infection (UTI) prevention recommends not screening for or treating ASB in catheterized patients.^6^ Despite these recommendations, unnecessary preoperative urine cultures remain prevalent.

Diagnostic stewardship optimizes diagnostic test use to prevent diagnostic error and reduce unnecessary downstream treatment including antibiotic overuse.^8^ Education combined with audit and evaluative feedback^9^ has demonstrated effectiveness in modifying clinical behavior and represents an evidence-based strategy for reducing low-value care, especially when paired with co-interventions such as interactive feedback and an actionable improvement plan.^10^

## Methods

We conducted a prospective pre/post quasi-experimental pilot study across four VAMCs randomly selected from a 15-site research consortium over the following periods: (1) preintervention, January 1 – December 31, 2022, (2) postintervention, January 1 – October 31, 2023. The intervention targeted patients in neurosurgery, orthopedic, and vascular surgery specialties.^2^ The multi-modal intervention comprised site-specific data feedback forms (intervention tool, Supplements A–C) and implementation activities (audit with interactive and evaluative feedback, and education). Our implementation approach was based on interviews with VA surgeons and other healthcare workers that identified audit and feedback combined with education as the most acceptable methods to reduce preoperative urine cultures.^11^ Feedback forms were developed by the central research team and delivered by local facility teams. They provided benchmarking data comparing urine culture rates by unit and specialty against consortium averages and summarized current evidence against preoperative urine cultures, with advice on additional infection prevention steps.

The intervention was implemented at each site by multidisciplinary local facility teams. Each local team included a site-based study co-investigator as study champion (e.g., hospital epidemiologist). Local teams used facility-level knowledge for targeted audit, feedback and education communication with surgical practice leaders and some individual high utilizers. The implementation activities ranged from targeted data review with surgical specialty leadership to general education for surgical services (e.g., in surgical meetings) using the feedback forms.

We collected data on all urine cultures obtained within 30 days before scheduled surgery on asymptomatic patients, excluding cultures performed for acute infectious symptoms (e.g., cultures collected in emergency departments or from febrile patients (temperature > 38°C) or from patients with urinary symptoms identified via manual chart review).^1^

The outcome was the proportion of surgeries with preoperative urine cultures. We compared rates between preintervention and postintervention periods, calculating preoperative urine culture rates by facility and surgical specialty. Statistical significance was assessed using χ^2^ tests. Analysis was performed using R, version 4.0.5 (R Core Team) and Microsoft Excel.

Of note, two intervention VAMCs (VAMC 2 and 4) already had diagnostic stewardship strategies in place to reduce unnecessary urine cultures and were involved in a study piloting additional strategies to reduce inpatient antibiotic use for UTI during our intervention period.^12^ The additional strategies piloted did not specifically target preoperative urine culturing, did not measure outpatient urine cultures, and were not uniformly effective in reducing urine cultures.^12^ This research was approved by the VA Central IRB and each VAMC’s research and development committee.

## Results

Overall, 5,508 surgeries eligible for inclusion were performed at the four intervention VAMCs across the study period, with 952 preoperative urine cultures eligible for inclusion. There was substantial variation in preoperative urine culturing by VAMC and surgical specialty (Table 1). Preintervention, rates ranged from 1% to 79% for neurosurgery, 1% to 52% for orthopedic surgery, and 4% to 19% for vascular surgery.

**Table 1.** Preoperative urine culture rates by facility and surgical specialty Table 1 long description.

	Urine cultures						
	Pre-intervention			Post-intervention			Absolute change (percentage points)
Site/Specialty	N cultures	*N* eligible surgeries	%	*N* cultures	*N* eligible surgeries	%	
***VAMC 1***							
Orthopedic	230	446	52	35	287	12	−40%*
Neurosurgery	168	214	79	99	133	75	−4%
Vascular	11	267	4	9	186	5	+1%
***VAMC 2***							
Orthopedic	95	345	28	57	325	18	−10%*
Neurosurgery	50	117	43	21	75	28	−15%
Vascular	7	114	6	7	57	12	+6%
***VAMC 3***							
Orthopedic	9	625	1	9	521	2	+1%
Neurosurgery	1	134	1	4	128	3	+2%
Vascular	49	217	23	53	178	30	+11%
***VAMC 4***							
Orthopedic	12	433	3	10	361	3	0%
Neurosurgery	3	93	3	3	77	4	+1%
Vascular	7	102	7	3	73	4	−3%
**Cumulative results from all sites**	20.7%			12.9%			−7.8%

* Statistically significant differences (*P* < .05).

**Figure 1.**
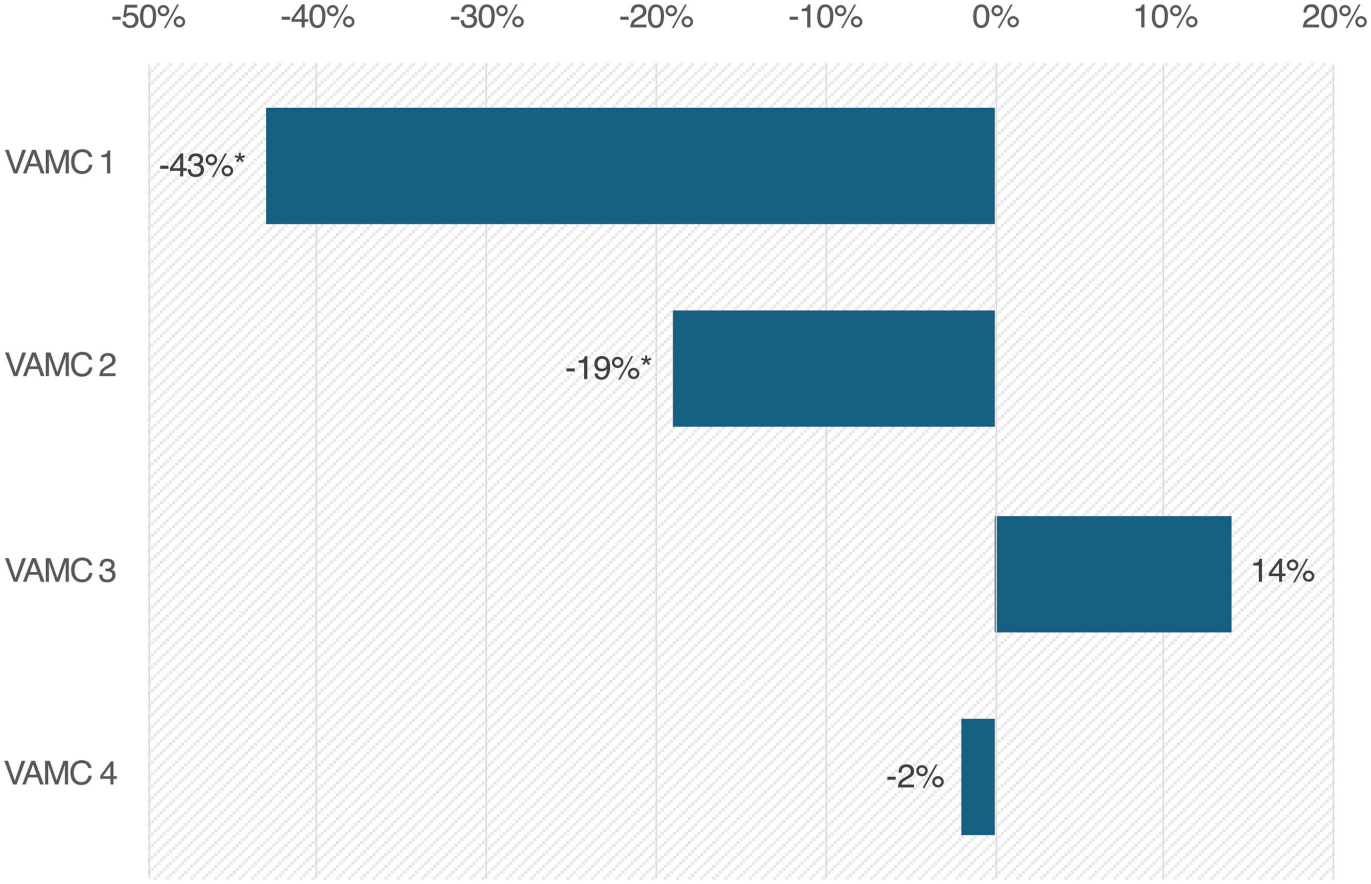
Figure 1 long description. Change in percentage urine culture postintervention at each site. * Statistically significant change in percentage (*P* < .05).

In total, preoperative urine culture rates decreased from 20.7% in the preintervention period to 12.9% in the postintervention period, representing a 37.5% relative reduction (95% confidence interval [95%CI: 29.2%–44.8%]; *P* < .01). Two VAMCs demonstrated statistically significant declines in urine culturing (Figure 1).

Orthopedic surgery demonstrated the largest overall reductions, though results varied considerably between facilities. VAMC 1 showed the most dramatic reduction in orthopedic surgery urine cultures (52% to 12%, 76.3% relative reduction, [95%CI: 67.4%–82.9%]; *P* < .01]). VAMC 2 showed a reduction in neurosurgery (43% to 28%, 34.5% relative reduction, [95% CI: .36%–56.9%]; *P* = .05]) and a significant reduction in orthopedic surgery (28% to 18%, 36.3% relative reduction, [95%CI: 14.8%–52.4%]; *P* < .01). Some surgical specialties experienced small, non-statistically significant increases in urine testing. VAMCs 3 and 4 had relatively low baseline rates and showed minimal changes.

## Discussion

Our multi-center study demonstrates that education combined with audit and feedback may reduce unnecessary preoperative urine culture testing. The 37.5% overall relative reduction represents progress toward evidence-based preoperative care and supports the potential for similar diagnostic stewardship strategies.

The substantial variation in preintervention urine culture utilization among facilities and surgical specialties suggests that usage is driven primarily by individual providers and local contextual factors, rather than specialty-specific or nationwide policies. Differences among sites may also reflect independent VAMC efforts to decrease utilization. VAMC 2 and 4 each had additional strategies implemented as part of quality improvement or research and these had variable results in terms of urine culture rates and antibiotic use rates. These additional strategies did not lead to uniform reductions across specialties. This underscores our findings that interventions need to be tailored to the specific site/unit/specialty to have the most efficient effect.^12^ These concurrent activities and the heterogeneity associated with a tailored intervention are limitations of our work, but reflect the real-world conditions of complex academically-affiliated medical centers.

Our results also demonstrate ceiling effects of low baseline usage: at VAMCs 3 and 4, preintervention rates were low, such that further intervention produced no statistically significant changes. Urine culture persistence at the lowest-utilization site postintervention suggests that some cultures remain clinically indicated (e.g., symptoms of UTI). Another limitation is that our research focused solely on process rather than clinical outcomes. Yet, prior studies have consistently shown that preoperative ASB is not associated with postoperative surgical site infections or UTI.^1^

Orthopedic surgery showed the greatest response to intervention, potentially reflecting education on recent evidence against routine preoperative urine cultures in joint replacement surgery, and opportunities for focused educational messaging around prosthetic joint infection prevention. Variation in neurosurgery and vascular surgery likely reflects differences in baseline knowledge, guideline interpretation, and local surgical culture, underscoring the importance of targeted strategies.

These findings support the effectiveness of targeted strategies for reducing low-value preoperative testing, when delivered via local clinical champion engagement. Observed variation between facilities highlights the need for flexible implementation approaches adaptable to local contexts and practice patterns. Advantages include adaptability to specialty, and the potential to spread knowledge to trainees and colleagues (train the trainer effect).

## Conclusion

This pilot intervention of education combined with interactive audit and evaluative feedback may have reduced unnecessary preoperative urine culture testing across multiple VAMCs. Substantial variation in intervention effectiveness between facilities and surgical specialties emphasizes the importance of tailored diagnostic stewardship strategies.

## Supporting information

10.1017/ice.2026.10470.sm001 Parmasad et al. supplementary material 1 Parmasad et al. supplementary material

10.1017/ice.2026.10470.sm002 Parmasad et al. supplementary material 2 Parmasad et al. supplementary material

10.1017/ice.2026.10470.sm003 Parmasad et al. supplementary material 3 Parmasad et al. supplementary material

## Supplementary material

The supplementary material for this article can be found at https://doi.org/10.1017/ice.2026.10470

## Table 1. Long description

The table presents data on preoperative urine culture rates by facility and surgical specialty across four Veterans Affairs Medical Centers (VAMCs) before and after an intervention. It includes columns for the number of urine cultures, eligible surgeries, and the percentage of cultures pre- and post-intervention, along with the absolute change in percentage points. The table is divided into sections for each VAMC, with rows for orthopedic, neurosurgery, and vascular specialties. Notable trends include a significant reduction in urine culture rates for orthopedic surgery at VAMC 1 and VAMC 2, while neurosurgery and vascular surgery show varied changes across different VAMCs. The cumulative results from all sites indicate an overall decrease in urine culture rates post-intervention.

Navigate back to Table 1..

## Figure 1. Long description

The horizontal bar graph compares the percentage change in urine culture post-intervention at four sites. The x-axis ranges from negative fifty percent to positive twenty percent, with increments of ten percentage points. The y-axis lists four sites labeled VAMC 1, VAMC 2, VAMC 3, and VAMC 4. VAMC 1 shows a significant decrease of forty-three percent, marked with an asterisk indicating statistical significance. VAMC 2 shows a decrease of nineteen percent, also marked with an asterisk. VAMC 3 shows an increase of fourteen percent, and VAMC 4 shows a slight decrease of two percent. The bars are colored in dark blue, and the values are clearly labeled on each bar. All values are approximated.

Navigate back to Figure 1..
