# Supplementary material for: An education with audit and targeted feedback intervention to de-implement preoperative surgical urine cultures: a multi-center quasi-experimental study
Source: Infect Control Hosp Epidemiol. 2026 Jun 5;47(7):748–51. doi: 10.1017/ice.2026.10470 (PMC13315520; doi:10.1017/ice.2026.10470)
Supplement: Parmasad et al. supplementary material 1 — Parmasad et al. supplementary material [file S0899823X2610470Xsup001.pdf]

De-Implementation of the Culture of Culturing Pilot Study

Our VA Medical Center was randomized to be a research site to test the effect of an intervention to reduce pre-op urine cultures among asymptomatic Veterans.

Background: The Infectious Diseases Society of America recommends against screening for asymptomatic bacteriuria among patients undergoing elective non-urologic surgery.

- *2019 Clinical Practice Guideline for the Management of Asymptomatic Bacteriuria*

This research team found that among asymptomatic VA surgical patients, pre-operative urine cultures +/- antibiotics did not change post-surgical outcomes regardless of surgical type or use of implants

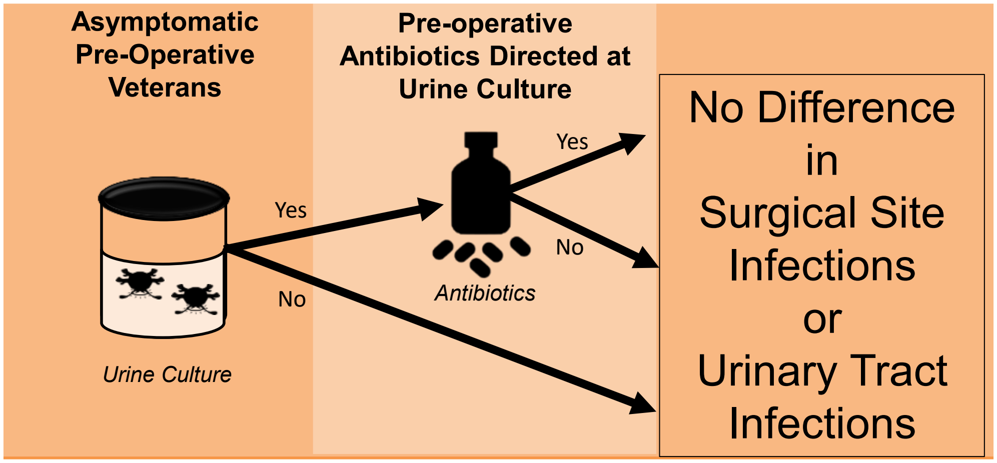

Gallegos Salazar et al. *JAMA Surg 2019*.

O'Brien et al, Under Review

Compared with other academically affiliated VA hospitals (blue), VAMC 4 (red) performs more pre-op urine cultures. This is getting worse over time.

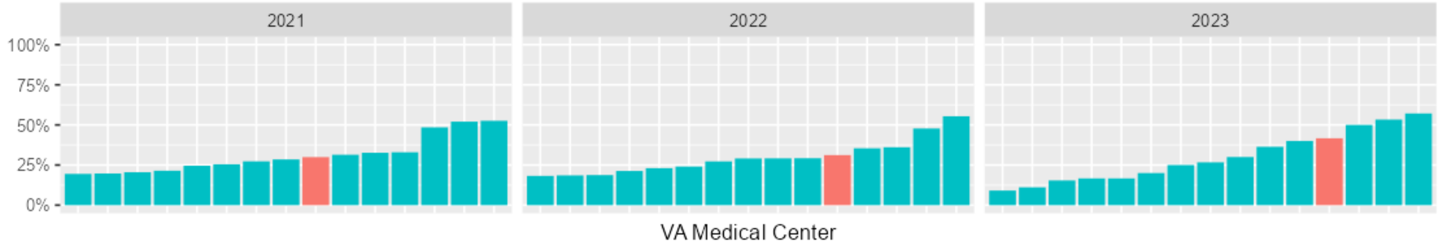

Each month, the research team will determine which pre-op cultures were performed among asymptomatic Veterans. We will reach out to clinicians or surgical leadership to work together to determine how to perform fewer future urine cultures among asymptomatic Veterans. This may include:

- A review of the evidence to reassure the clinician that urine culture is not needed
- Removal of the test from order sets
- New education for incoming surgical residents
- Other interventions determined by surgical teams

For more information, please contact the local site PI.
