# Supplementary material for: An education with audit and targeted feedback intervention to de-implement preoperative surgical urine cultures: a multi-center quasi-experimental study
Source: Infect Control Hosp Epidemiol. 2026 Jun 5;47(7):748–51. doi: 10.1017/ice.2026.10470 (PMC13315520; doi:10.1017/ice.2026.10470)
Supplement: Parmasad et al. supplementary material 2 — Parmasad et al. supplementary material [file S0899823X2610470Xsup002.pdf]

Changing the Culture of Culturing:
De-Implementing Testing and Treating of Urine Cultures in Asymptomatic Patients
Co-PIs: Kalpana Gupta, MD and Marin Schweizer, PhD

Project goal: Reduce inappropriate urine cultures in asymptomatic patients undergoing surgery

Why? Research demonstrates that urine cultures done before surgery, ***without specific indication,*** show:

- ➔ No reduction in post-op UTI
- ➔ No reduction in post-op SSI
- ➔ Increased unnecessary antibiotics and increased C. diff

<u>VAMC 1</u>: In 2022-2023, 86% of neurosurgery patients had urine cultures done before surgery

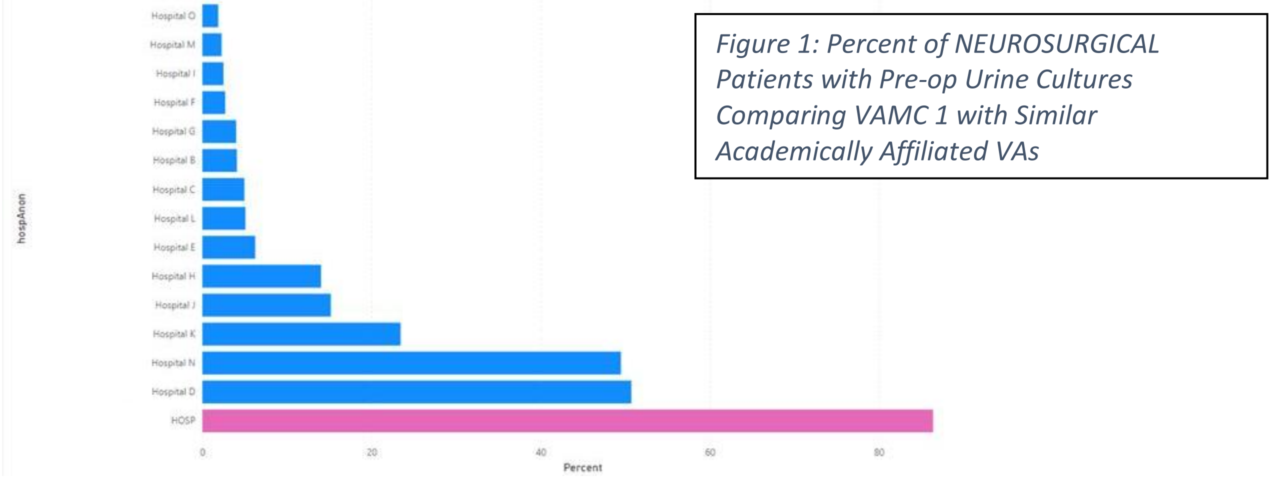

Figure 1: Percent of NEUROSURGICAL Patients with Pre-op Urine Cultures Comparing VAMC 1 with Similar Academically Affiliated VAs

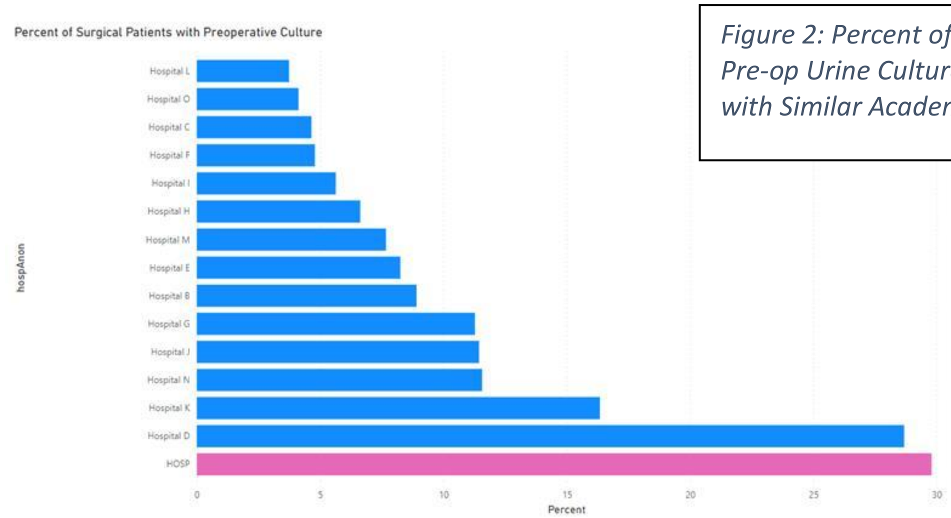

Figure 2: Percent of Surgical Patients with Pre-op Urine Cultures Comparing VAMC 1 with Similar Academically Affiliated VAs

Neurosurgery:

- <u>No reduction in SSI or UTI by doing a preoperative screening urine or treating asymptomatic positives</u>. JAMA Surgery 2018 VA Boston
- <u>Improving Value of Care: Cessation of Screening Urine Culture Prior to Orthopedic and Spinal Surgery</u>. Mayo Clinic Proceedings. Walter C. Hellinger et al

  An 87% (153 vs 1141) reduction in screening urine cultures over a 12-month period was associated with a reduction of 988 unnecessary urine cultures, an 83% (6 vs 35) decline in inappropriate antibiotic treatment of asymptomatic bacteriuria, and no increase in SSI incidence after hip replacement, knee replacement, spinal fusion, or laminectomy procedures.

Next steps:

- ➔ Forget the urine; focus on standard skin antisepsis measures (CHG; MRSA detection)
- ➔ Avoid foleys; maintain good urinary flow
