# Supplementary material for: An education with audit and targeted feedback intervention to de-implement preoperative surgical urine cultures: a multi-center quasi-experimental study
Source: Infect Control Hosp Epidemiol. 2026 Jun 5;47(7):748–51. doi: 10.1017/ice.2026.10470 (PMC13315520; doi:10.1017/ice.2026.10470)
Supplement: Parmasad et al. supplementary material 3 — Parmasad et al. supplementary material [file S0899823X2610470Xsup003.pdf]

# Changing the Culture of Culturing:
## De-Implementing Testing and Treating of Urine Cultures in Asymptomatic Patients
Co-PIs: Kalpana Gupta, MD and Marin Schweizer, PhD

**Project goal:** Reduce inappropriate urine cultures in asymptomatic patients undergoing surgery

**Why?** Research demonstrates that urine cultures done before surgery, ***without specific indication,*** show:

- ➔ No reduction in post-op UTI
- ➔ No reduction in post-op SSI
- ➔ Increased unnecessary antibiotics and increased C. diff

**<u>VAMC 3</u>: Over the past 2 months, x (#) patients had urine cultures done before surgery <u>without</u> specific indication.**

**Figure: Annual trends in urine cultures at the Xxxx VA by surgery type, 2018-2023**

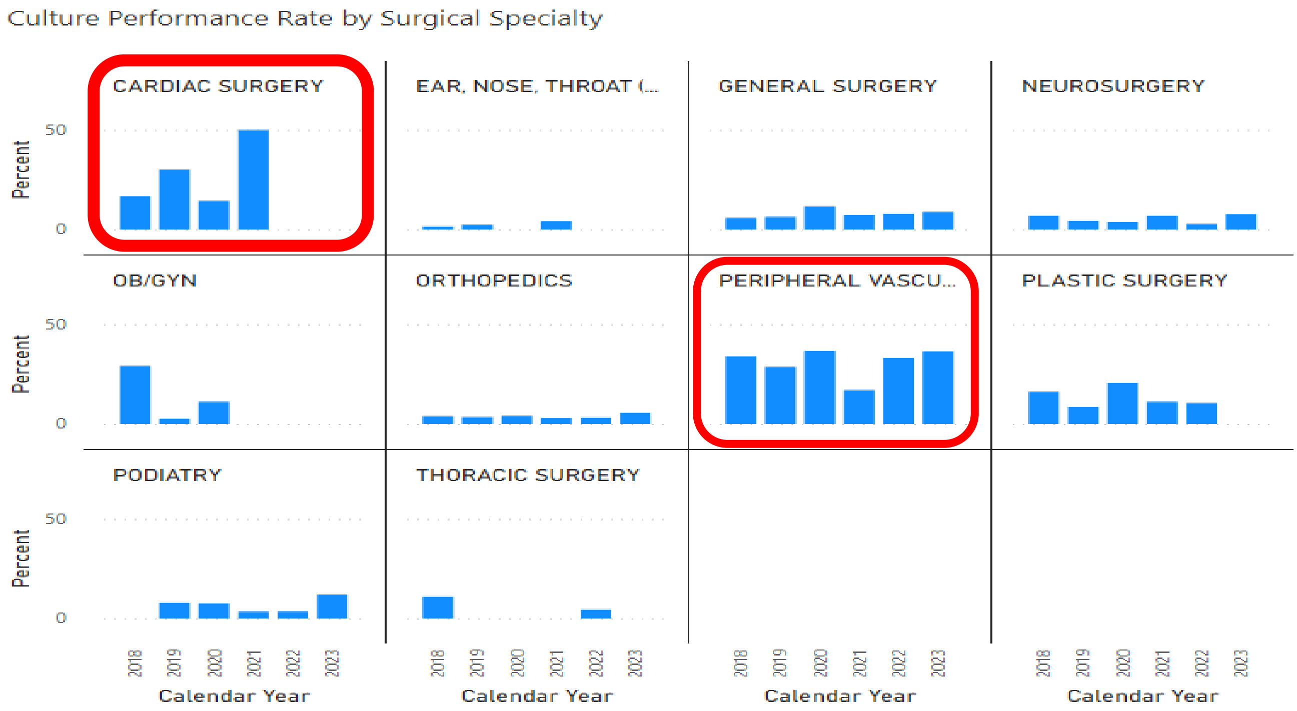


**Cardiac Surgery:**

- <u>Finding and Treating bacteriuria preoperatively does not reduce SSI risk</u> (JAMA Surgery 2018)
- *S. aureus* was the only pathogen that matched in urine and cardiac SSI

***Next steps***:

- ➔ Forget the urine; focus on standard skin antisepsis measures (CHG; MRSA detection)
- ➔ Avoid foleys; maintain good urinary flow

**Vascular Surgery:**

- <u>No reduction in SSI or UTI by doing a preoperative screening urine or treating asymptomatic positives</u> (JAMA Surgery 2018)
